# Phenotypic Robustness of Epidermal Stem Cell Number in *C. elegans* Is Modulated by the Activity of the Conserved N-acetyltransferase *nath-10*/NAT10

**DOI:** 10.3389/fcell.2021.640856

**Published:** 2021-05-18

**Authors:** Mark Hintze, Dimitris Katsanos, Vahid Shahrezaei, Michalis Barkoulas

**Affiliations:** ^1^Department of Life Sciences, Imperial College, London, United Kingdom; ^2^Department of Mathematics, Imperial College, London, United Kingdom

**Keywords:** developmental robustness, seam cells, epidermis, stem cells, *C. elegans*, nath-10, NAT10, Wnt signaling

## Abstract

Individual cells and organisms experience perturbations from internal and external sources, yet manage to buffer these to produce consistent phenotypes, a property known as robustness. While phenotypic robustness has often been examined in unicellular organisms, it has not been sufficiently studied in multicellular animals. Here, we investigate phenotypic robustness in *Caenorhabditis elegans* seam cells. Seam cells are stem cell-like epithelial cells along the lateral edges of the animal, which go through asymmetric and symmetric divisions contributing cells to the hypodermis and neurons, while replenishing the stem cell reservoir. The terminal number of seam cells is almost invariant in the wild-type population, allowing the investigation of how developmental precision is achieved. We report here that a loss-of-function mutation in the highly conserved N-acetyltransferase *nath-10/*NAT10 increases seam cell number variance in the isogenic population. RNA-seq analysis revealed increased levels of mRNA transcript variability in *nath-10* mutant populations, which may have an impact on the phenotypic variability observed. Furthermore, we found disruption of Wnt signaling upon perturbing *nath-10* function, as evidenced by changes in POP-1/TCF nuclear distribution and ectopic activation of its GATA transcription factor target *egl-18*. These results highlight that NATH-10/NAT-10 can influence phenotypic variability partly through modulation of the Wnt signaling pathway.

## Introduction

Developing organisms face a constant challenge from external (environmental) and internal (genetic) perturbations or stochastic noise in molecular processes. It is therefore remarkable that organisms can produce consistent phenotypes across populations in spite of these perturbations (Bauer et al., 2015; Felix and Barkoulas, 2015). Robustness, first described by Waddington (1959), is the term used to define the invariance of a phenotype when faced with incoming variation (Felix and Barkoulas, 2015). Robustness to various perturbations is particularly important in developing tissues, where cell division and differentiation patterns need to be tightly controlled to generate the required structures and cell types.

Phenotypic robustness has been mostly investigated in single cell organisms such as *Saccharomyces cerevisiae*, where for example more than 300 genes have been found to modulate morphological variation and suggest a genetic basis for phenotypic robustness (Levy and Siegal, 2008). However, robustness in multicellular animals is much less studied, so it remains a challenge to identify what are the buffering mechanisms in biological systems, as well as their sensitivities across cells and tissues for different perturbations (Felix and Barkoulas, 2015). For example, a recent study in *Drosophila melanogaster* has shown that neuronal wiring in the thorax is highly variable to genetic and environmental pressure and this variability is under the control of the Hox gene *Ultrabithorax* (Mellert et al., 2016), suggesting that individual genes can buffer or enhance variability in higher organisms depending on the developmental context.

**FIGURE 1:**
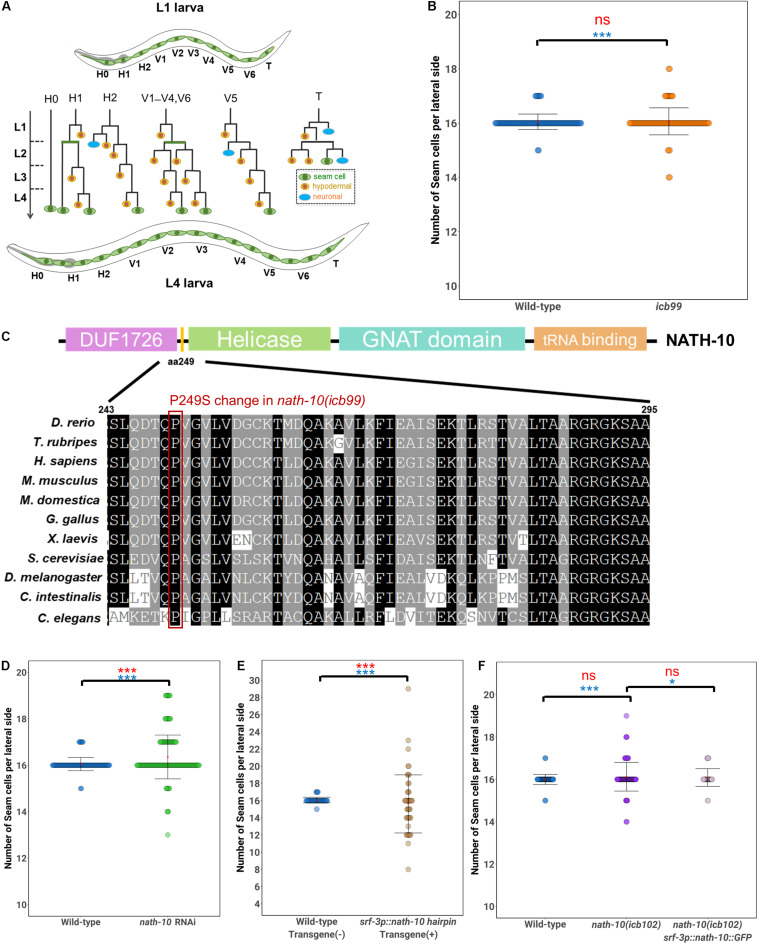
Seam cell number robustness is disrupted by *nath-10* loss-of-function. **(A)** schematic displaying seam cell lineage from L1 to L4, seam cells are labeled green, hypodermal cells are labeled yellow and neurons are labeled blue. **(B)** Mutants carrying the *icb99* mutation display variable seam cell number (*SD* = 0.28, *n* = 120 for wild type JR667 animals, *SD* = 0.50, *n* = 120 for animals carrying the *icb99* mutations. Red ns indicates non-significant difference to the mean by *t*-test (*p* > 0.05). Blue stars indicate statistically significant difference to the variance by Levene’s median test (***p < 0.001). **(C)** Schematic showing protein domains in NATH-10. The P249S change involves a proline to serine shift at amino acid 249, indicated by the yellow bar between the DUF1726 and Helicase domains. Alignment of this region across multiple species shows high conservation of this proline, suggesting it may be of importance to protein function. Red frame shows the conserved proline. **(D)** *nath-10* RNAi phenocopies the *icb99* mutation leading to a significant increase in variance and average seam cell number (Levene’s median test ***p < 0.001 and *t*-test, ***p < 0.001, *n* = 120 wild type, and *n* = 183 *nath-10* RNAi). **(E)** Seam cell specific knockdown of *nath-10* by a *nath-10* hairpin RNAi also causes significant increase in seam cell number and seam cell variance in comparison to wild-type animals not carrying the hairpin transgene (*t*-test, ***p < 0.001, Levene’s test median ***p < 0.001). **(F)** A CRISPR replacement mimicking the *icb99* mutation phenocopies the original EMS-derived mutant (*SD* = 0.77, Levene’s test ***p < 0.05). Seam cell specific overexpression of *nath-10* (*srf-3p:nath-10:GFP*) partially rescues this phenotype (*SD* = 0.41, Levene’s test *p < 0.05 In **(B,D–F)** mean values are indicated by red dots and error bars correspond to ± Standard deviation. Statistical differences to the mean and variance are indicated above brackets for relevant comparisons in red and blue, respectively, assessed by a *t*-test or Levene’s median test. Significance levels (ns *p* > 0.05, *p < 0.05, ***p < 0.001).

*C. elegans* is a highly tractable model for investigating phenotypic robustness due to its well-established cell lineage, fully characterized genome, and isogenic nature, which leads to little genetic variation within a population (Sulston and Horvitz, 1977). Lack of genetic variation removes a key confounder when it comes to studying robustness in phenotypic variation at the population level. Here, we focus on a population of cells in the developing larva that have stem cell properties, the seam cells. Seam cells are found on the lateral sides of the animal in two lines running from anterior to posterior and are labeled as H0-H2 (Head), V1-V6 (Ventral), and T (Tail) (Chisholm and Hsiao, 2012). Seam cells go through rounds of stem cell-like asymmetric and symmetric divisions during larval development. Animals hatch at L1 with 10 seam cells and these expand through a round of symmetric division at the L2 larval stage to a final number of 16. Furthermore, reiterative asymmetric cell divisions throughout larval development contribute the majority of nuclei to the developing hypodermal syncytium and a number of neurons (Figure 1A). In this context, we have recently conducted a mutagenesis screen to find mutations that introduce stochastic variability in terminal seam cell number (Katsanos et al., 2017). A proof-of-concept was established showing that mutations in the Hes-related basic helix-loop-helix transcription factor *lin-22* increase seam cell number variability. Loss of seam cells early in post-embryonic development due to ectopic neurogenesis were found to be compensated later in larval development via stochastic symmetrization of cell divisions, thereby causing the seam cell number to be distributed on either side of the mean.

A number of other transcription factors and signaling pathways have been found to regulate seam cell development. GATA transcription factors, such as *elt-1* and *egl-18*, the engrailed homolog *ceh-16* and the *Runx*/*CBF*β homologs *rnt-1*/*bro-1* are thought to be key players in the seam cell gene network (Smith et al., 2005; Huang et al., 2009; Gorrepati et al., 2013; Gorrepati and Eisenmann, 2015). During seam cell patterning, the Wnt pathway activates targets necessary for seam cell maintenance, such as *egl-18*, through the downstream effector POP-1/TCF, while ectopic *egl-18* activation can drive ectopic seam cell fate retention (Gorrepati et al., 2013). Activation of Wnt signaling in one of the two daughter cells relies on asymmetric inheritance of Wnt pathway components from polarized mother cells (Mizumoto and Sawa, 2007), while this is overridden during symmetric cell divisions through the repression of *pop-1/*TCF by the RNT-1/BRO-1 module (Yamamoto et al., 2011; Hughes et al., 2013; van der Horst et al., 2019). Other pathways, such as the heterochronic pathway, control developmental timing of the division patterns (Ambros and Horvitz, 1984; Harandi and Ambros, 2015).

Here, we continue the characterization of mutations from our genetic screen and demonstrate that a mutation in the highly conserved N-acetyltransferase *nath-10* disrupts seam cell number robustness. We report that the defect in phenotypic variance is restricted to seam cells and may be associated with an increase in mRNA transcript variance among animal populations. We also demonstrate changes in the distribution of POP-1 and subsequent activation of the Wnt target *egl-18* upon impairment of *nath-10* function. Therefore, we propose that NATH-10 modulates robustness of seam cell patterning through regulation of the Wnt signaling pathway.

## Materials and Methods

### *C. elegans* Culture and Strains

*C. elegans* strains used in this study were maintained and raised according to standard protocols on NGM plates with the *E. coli* strain OP50 as a food source (Brenner, 1974). Wild-type animals referred to in this work are the strain JR667 (*wIs51*) that contains the transgene *scm:GFP.* A full list of strains used in this study can be found in Supplementary Table 1.

### Genetic Screening and Mapping

Mutagenesis of animals using EMS was carried out according to standard protocols (Brenner, 1974). The screen for terminal seam cell number mutants was carried out as described in Katsanos et al. (2017). Whole-genome sequencing was performed using an Illumina platform to reach 20–30-fold genome coverage and mapping was performed using the Cloudmap pipeline on a local version of Galaxy (Minevich et al., 2012). The mutation in the *icb99* allele is a C > T change in the third exon of *nath-10* (GAGACAAAA**C**CAATCGGACCATTGC) and it was backcrossed 4 times before phenotypic characterization.

### Microscopy and Phenotypic Characterization

Animals were mounted under a coverslip on 3% agarose in a droplet of M9 containing 100μM sodium azide for light and fluorescent microscopy. Seam cells on one lateral side were visualized using a Zeiss Compound microscope (AxioScope A1) at 40x and 100x at 44–48 h of development. Lineage analysis was carried out on bleach-synchronized animals carrying the *scm:GFP* transgene at 10, 17, 24, and 27 h post bleaching. Brood size assays were carried out by bleach synchronizing animals and then isolating single L4s. Single L4s were transferred to new plates every day for 5 days until no evidence of egg laying could be seen and the resulting progeny on each plate was counted. Fluorescence intensity for the *egl-18*:cherry and POP-1:GFP reporters were calculated in the following manner using ImageJ. A region of interest was drawn around seam cell nuclei and measurements of area and integrated density were taken. Three background readings were taken from areas surrounding the seam cells. Corrected total fluorescence was calculated with the following formula: Integrated Density – (Area (ROI) X Mean fluorescence of background readings.

### CRISPR-Cas9 Mediated Genome Editing

The single point mutation from the *nath-10(icb99)* allele was engineered into an N2 background via injection of Cas9 ribonucleoprotein complexes (Paix et al., 2015) in 10 μl as follows: tracRNA (0.75 nmol – IDT), custom crRNA (*nath-10* target sequence CGA GAA AGC AAT GGT CCG AT, IDT) and CAS9 (1 μg/μl -IDT). The co-injection markers *myo-2:dsRED* at 5 ng/μl and *rol-6*(*su1006)* at 40 ng/μl were used to select transgenic animals, and a repair template (6 μM) that introduced a *Cla*I restriction site. Therefore, *nath-10(icb102)* contains the point mutation found in *nath-10(icb99)* and a synonymous mutation that introduces a *Cla*I site. F1 animals were screened for the co-injection markers, allowed to lay progeny, and screened using a restriction digest for the introduced *Cla*I site. Positive lines were Sanger-sequenced to identify animals homozygous for SNP insertions.

### Molecular Cloning

To produce *srf-3p:nath-10:GFP*, *nath-10* was amplified from N2 cDNA using primers hN53 and hN54, which carried compatible sequences allowing insertion to the *Xma*JI/*Pac*I digested pDK52 via Gibson assembly creating pHIN9 (*srf-3p:nath-10:GFP:unc-54- 3′UTR).* The *srf-3* promoter used in this study refers to the first intron of the *srf-3* locus fused to a minimal *pes-10* promoter which drives expression specifically in seam cells.

To produce a platform for quick and efficient assembly of hairpin-RNAi constructs from a single PCR product by golden gate assembly, the following constructs were made. The plasmid pDK102 carrying a *dpy-7* promoter and the *p10 3′UTR* (Pfeiffer et al., 2012) in a pCFJ151 backbone was digested with *Xma*JI/*Pac*I to linearize and allow for cloning between the promoter and the 3′UTR. A gene fragment (Golden Gate Hairpin) carrying a compatibility arm to the *dpy-7* promoter, an outron, 2 inverted repeats of the *Bpi*I enzyme the 5th intron from the *srf-3* gene, two inverted repeats of the *Esp*3I enzyme and compatibility arm to the *p10 3′UTR* was synthesized (GENEWIZ) and was inserted in the digested pDK102 to form the intermediate plasmid pDK109 (*dpy-7p::Golden Gate Hairpin:p10 3′UTR* + *cb-unc-119*). Using oligos DK186 and DK179 the sequence from the promoter to the 3′UTR was amplified and inserted by Gibson assembly in a *Kpn*I/*Not*I digested pBluescript vector to form pDK110 (*dpy-7p::outron::GGBpiI::srf-3a intron5::GGEsp3I::p10 3UTR*). To modify the golden gate (GG) enzyme sites such that they leave non-palindromic scars to allow for specific directional cloning the *srf-3* intron 5 was amplified from pDK110 using oligos DK212 and DK214. The resulting amplicon was amplified with oligos DK213 and DK215 and was inserted in a *Bpi*I/*Esp*3I digested pDK110 backbone by Gibson assembly to produce pDK127 (*dpy-7p::outron::non-palGGBpiI::srf-3a intron5::non-palGGEsp3I::p10 3′UTR*).

To design a GFP hairpin, a fragment from GFP not containing sites for *Bpi*I and *Esp*3I was amplified from L3135 using oligos DK203 and DK204 and was inserted by Golden gate assembly in pDK127 to create pDK130(*dpy-7p::outron::* > *GFP-frag* > *::srf-3a intron5::* < *GFP-frag* < *::p10 3UTR*). To create a seam cell expressing version the *srf-3p* promoter was amplified from pDK126 using oligos DK234 and DK244 and was inserted in a Gibson assembly reaction with *Sal*I digested pDK130 to remove the *dpy-7* promoter and create pDK134(*srf-3p::outron::* > *GFP-frag* > *::srf-3a intron5::* < *GFP-frag* < *::p10 3UTR*). For pHIN33, 366 bp from the first exon of *nath-10* was amplified using the primers hN79 and hN80 with complementary tags which allowed golden gate assembly into a *Bpi*I and *Esp*3I digested pDK157. Golden gate assembly reactions consisted of adding 2.5 units of T4 DNA Ligase (Thermo Fisher Scientific), 0.5 μl of the *Bpi*I and 0.5 μl of the *Esp*3I FastDigest enzymes (Thermo Fisher Scientific), 50 ng of the vector plasmid and a 2::1 insert:: vector molar ratio in 1x T4 Ligase buffer (Thermo Fisher Scientific) in a total volume of 10 μl. Reactions were incubated at 37°C for 30 min, 5 min at 50°C and 5 min at 80°C in a thermocycler. All plasmids were verified by Sanger sequencing. A complete list of plasmids and oligos is presented in Supplementary Table 2.

### Single Molecule Fluorescence *in situ* Hybridization

Bleach-synchronized populations of animals were fixed at the appropriate stage as directly monitored by microscopy, smFISH was performed as previously described (Barkoulas et al., 2013) using a pool of 24–48 oligos fluorescently labeled with Cy5 (Biomers, Germany). Imaging was performed using a motorized epifluorescence Ti-eclipse microscope (Nikon) and a DU-934 CCD-17291 camera (Andor Technology, Belfast, United Kingdom) acquiring 0.8 um step z-stacks. Image analysis and spot quantification were performed on raw data using a MATLAB (MathWorks, Natick, MA) routine as previously described (Barkoulas et al., 2013), with the addition of a step to optimize ROI size on the Z-axis in adjacent sections within the area of interest. For images presented in the results section of this study, the probe signal channel was inverted for clarity (black spots correspond to mRNAs) and merged to the seam cell fluorescent marker in ImageJ (NIH, Rockville, MD). A complete list of smFISH oligo probes is presented in Supplementary Table 2.

### RNA Sequencing Analysis

Larvae were synchronized by bleaching from 6 plates per replicate and grown to L2 stage (27 h post hatching). The same timepoint was used for RNA collection for wild type and *nath-10* mutants since we verified by monitoring seam cell divisions that the animals were growing at the same speed. We used 3 replicates for each condition (we estimate ∼5,000 animals per replicate) and total RNA was extracted using the TRIzol reagent (Invitrogen, Carlsbad, CA). Illumina sequencing was performed by BGI (China). The sequencing data were processed and aligned to the *C. elegans* reference genome using kallisto (Bray et al., 2016). The counts were normalized using the DESeq2 package in R (Anders and Huber, 2010). Differences in gene expression were calculated using the negative binomial test in the DESeq package (FDR = 0.1). Coefficient of variation was calculated by dividing the standard deviation of mean transcript number by the mean number of transcripts. Seam cell-enriched genes based on sci-RNA-seq (single-cell combinatorial indexing RNA sequencing) data were defined by removing genes with fewer than 10 transcripts per million across all tissue clusters, genes enriched only in the germline, as well as ubiquitously expressed genes across all tissue clusters. Seam cell-enriched genes were defined as having a count > 10 transcripts per million in the seam cell tissue cluster and on average less than 100 counts in other tissue clusters. These filtering steps gave rise to 6,014 genes in total. Functional subsets of genes were obtained by downloading gene expression clusters for these terms from Wormbase and WormMine. RNA-seq data are deposited in NCBI GEO under accession number GSE162226.

### RNAi by Feeding

Animals were fed with dsRNA expressing bacteria as a food source. Bacteria were grown over-night and then seeded directly onto NGM plates containing 1 μM IPTG, 25 μg/ml ampicillin and 6.25 μg/ml tetracycline. Young adults were bleached to synchronize the population and eggs were plated on RNAi or empty vector plates and counted seam cells at the late L4 stage the *nath-10* clone used in this study was obtained from the Ahringer library (Source BioScience).

### Statistical Analysis

All statistical analysis was carried out in the R statistical package (R Core Team, 2020) and all plots were carried out using ggplots2 (Wickham, 2016).

## Results

### A Forward Genetic Screen Identifies a Mutation in *nath-10* Affecting Seam Cell Number Robustness

To identify modulators of terminal seam cell number robustness, we carried out a forward genetic screen. We mutagenized L4 larvae carrying the transgene *scm::GFP* (*wIs51*) by treating them with EMS, allowed F1 animals to produce progeny and isolated F2 animals that had an extreme departure from the average terminal seam cell number of 16 cells per lateral side in wild type. Phenotypic extremes refer to individuals having either greater than 17 or less than 15 seam cells, which occur very infrequently (less than 1%) in the wild-type population. The selected F2 mutant animals could therefore display at the F3 population either an increase, decrease or variable terminal seam cell number. As seam cell number variance changes as a function of the average number of seam cells (Katsanos et al., 2017), we focused on animals that had an average of 16 seam cells but had a significant increase in variability (identified using a Levene’s test), usually with errors on both sides of the wild-type mean. One mutation that fitted these criteria was *icb99*, which led to a significant increase in the standard deviation of seam cell number compared to the wild type (Figure 1B).

To identify the molecular change in *icb99* we used mapping-by-sequencing (Minevich et al., 2012; Doitsidou et al., 2016). To this end, we crossed the mutant harboring *icb99* to the polymorphic *C. elegans* isolate CB4856. F2 progeny with a similar increase in phenotypic variance were isolated, pooled and whole-genome sequenced. This experiment identified a region on the left arm of chromosome I that contained preferentially N2 sequences. Within this region, we found a C to T change in the third exon of the N-acetyltransferase *nath-10*, which is the homolog of the human NAT10. This change results in a non-synonymous substitution of a proline to a serine (P249S). Although, this change is not within the known domains of NATH-10, the affected proline is conserved from yeast to humans, suggesting it may affect some structural feature of the protein (Figure 1C).

To validate that the *nath-10(icb99)* mutation was causative for the seam cell number variance phenotype, we first carried out RNAi against *nath-10* in the wild-type population. We found that *nath-10* RNAi affected seam cell patterning and significantly increased seam cell number variance (Figure 1D). However, *nath-10* RNAi also caused a high rate of sterility, as well as arrest in early larval development, phenotypes which were not observed in the recovered allele from our mutagenesis screen. This is consistent with previously reported *nath-10* loss-of-function phenotypes in *C. elegans*, such as lethality, sterility and defects in vulval development (Duveau and Félix, 2012).

To circumvent these pleiotropic effects and address whether the seam cell phenotype might be a response to global or seam cell-specific impairment of *nath-10* function, we knocked down *nath-10* in the seam cells using a *nath-10* hairpin. To this end, we devised an approach that facilitates cloning of any gene fragment in sense and antisense orientation under an epidermal promoter using a single-step golden gate assembly (Supplementary Figure 1A). Such transgenes result in the production of hairpin dsRNA structures that have been shown to cause silencing of target genes in multiple tissues (Tavernarakis et al., 2000; Timmons et al., 2003). To confirm that the system is effective, we performed control experiments targeting GFP expression with a GFP hairpin. Seam cell expression of a hairpin against GFP in animals expressing *scm::GFP* led to complete abolishment of GFP signal in 50% of the transgenic animals and strong observable reduction of signal in another 36.2% of animals (Supplementary Figure 1B). A similar effect was observed in the case of hypodermal (hyp7) expression of a GFP hairpin and a hypodermal GFP marker (*dpy-7p::GFP*), while hypodermal expression of the hairpin resulted in no reduction in the *scm::GFP* signal (Supplementary Figure 1B). Having established the functionality and specificity of the system, we produced transgenic animals harboring a seam cell driven *nath-10* hairpin. We found that seam cell specific knockdown of *nath-10* caused a significant increase in seam cell variance (Figure 1E), when compared to animals that did not carry the transgene and did not cause other phenotypes, such as larval lethality. These results therefore suggest that *nath-10* is likely to be the causative mutation and that *nath-10* may act in a cell autonomous manner to influence seam cell patterning.

To further validate the causative mutation underlying the increase in seam cell number variance, we generated by CRISPR editing a new allele (*icb102*) mimicking the nucleotide change found in *nath-10(icb99).* We found that the CRISPR allele phenocopied the original *icb99* allele with regard to seam cell number variance (Figure 1F). The increase in variance was again moderate, but it was very reproducible across different days of scoring (Supplementary Figure 2). Furthermore, by introducing *nath-10* under a seam cell specific promoter (*srf-3p::nath-10::GFP*) we could largely rescue the phenotype of *icb102* (Figure 1F). Taken together, these results indicate that a mutation in *nath-10* breaks down seam cell number robustness. It is of note that the newly recovered *nath-10* allele did not show other obvious developmental phenotypes and vulval cell fate errors, but did show a decrease in mean brood size, while the variance in progeny number was not statistically different compared to the wild type (Supplementary Figure 3A). These results suggest that the increase in phenotypic variance upon loss of *nath-10* function is likely to be specific to seam cells.

### The Mutation in *nath-10* Causes Gains and Losses in Seam Cells During Post-embryonic Development

**FIGURE 2:**
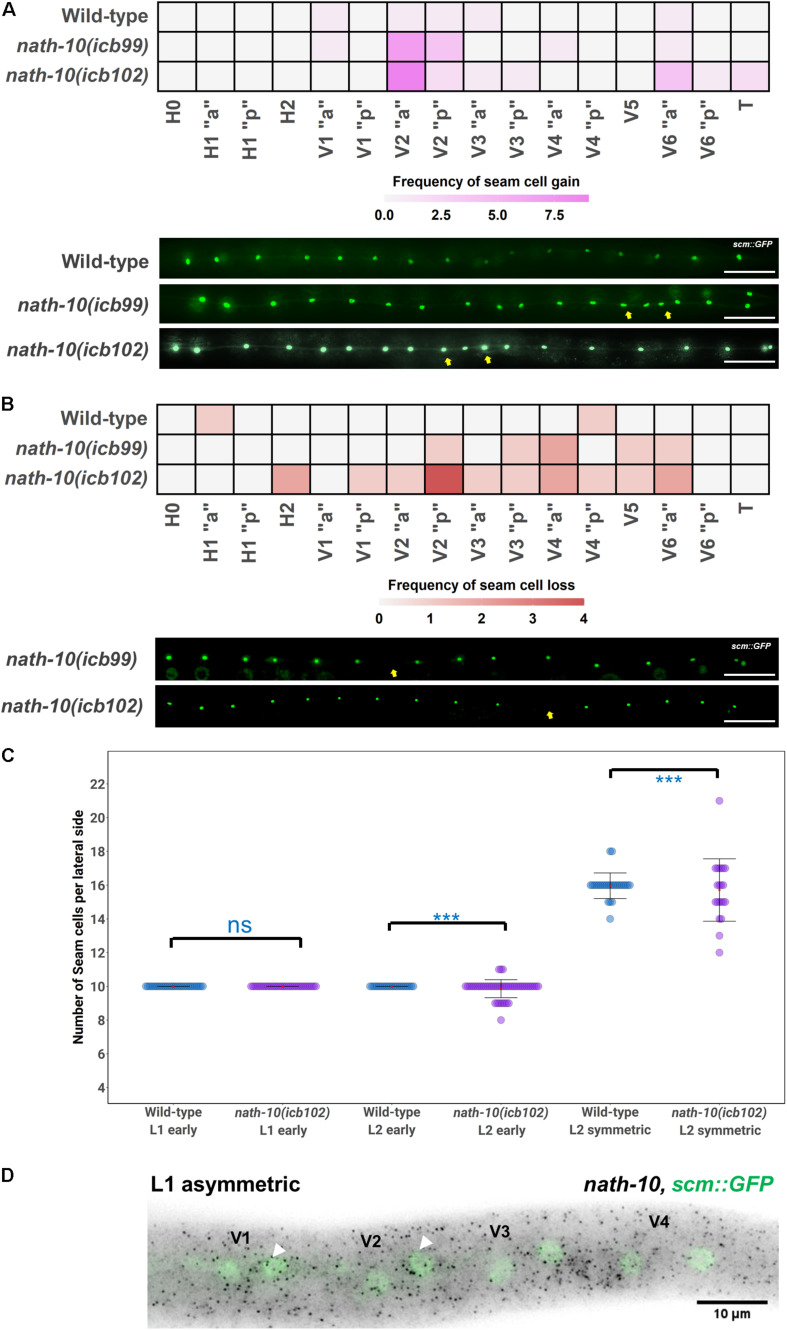
*nath-10* loss-of-function causes both gains and losses of seam cells from the early L2 stage. **(A)** Heat map displaying frequency of seam cell duplications for wild type, *nath-10(icb99)* and *nath-10(icb102)*. Increased seam cell duplication frequency occurs mostly in the V2 seam cell. Representative images below from wild type, *nath-10(icb99)* and nath-10(*icb102)* animals carrying the *scm::GFP* transgene. Yellow arrows indicate seam cell duplications. **(B**) Heat maps displaying seam cell losses in *nath-10* mutants. Note that losses are found more frequently in the V lineage seam cells. Yellow arrows indicate missing seam cells. In **(A,B)**, “a” and “p” represent the anterior and posterior branch of a seam cell lineage. **(C)** Seam cell counts at early L1, early L2 and mid L2 stage for wild type and *nath-10(icb102)* mutants. No difference was found at the early L1 stage as, all animals’ hatch with 10 seam cells, whereas a significant difference in variance of seam cell number was found at the L2 stage (Levene’s test ***p < 0.001). **(D)** Representative image of *nath-10* smFISH shows expression in seam cells at the time errors begin to occur (L1 division). White arrowheads point to posterior daughter cells following the L1 asymmetric division. Nuclei are labeled green because of s*cm::GFP* expression. Scale bars in **(A,B)** indicate 100 um.

Seam cells belong to well-described cell lineages and take stereotypic positions along the anterior-posterior axis (Figure 1A), allowing us to infer the origin of developmental defects based on changes in the terminal seam cell number observed in a mutant background. In *nath-10* loss-of-function mutants, we found that gains and losses of seam cells occur mostly in V2 and mid-body cell lineages (Figures 2A,B). We then sought to understand when such defects occur in development by carrying out seam cell counts at defined intervals. First, we ruled out embryonic defects and developmental delay phenotypes as both wild type and *nath-10(icb102)* mutants hatched with 10 seam cells (Figure 2C) and the proportion of animals reaching the L4 stage at defined time intervals was not significantly different compared to the wild type (Supplementary Figure 3B). However, we found that 10 h post hatching *nath-10(icb102)* animals began to show significant increase in seam cell number variability that became even more pronounced at the L2 stage (Figure 2C). These results suggest that *nath-10* mutation effects can be seen early in post-embryonic seam cell divisions.

To consolidate the lineaging and genetic results, we studied whether *nath-10* is expressed in the seam cells during development. To this end, we carried out *nath-10* single molecule fluorescence *in situ* hybridization (smFISH) and found expression in seam cells, for example in V cell lineages at the L1 asymmetric division stage (Figure 2D). The expression of *nath-10* was not specific to the seam, for example it was expressed in the germ line at the L4 stage (Supplementary Figure 3C), which is consistent with the observed brood size defects in *nath-10* alleles. Taken together, our results support the idea that *nath-10* disruption leads to seam cell number variability through both gains and losses of seam cells during early post-embryonic development and predominantly in the mid-body V lineages.

### RNA Sequencing Analysis Identifies an Increase in mRNA Transcript Number Variance in *nath-10* Mutants

**FIGURE 3:**
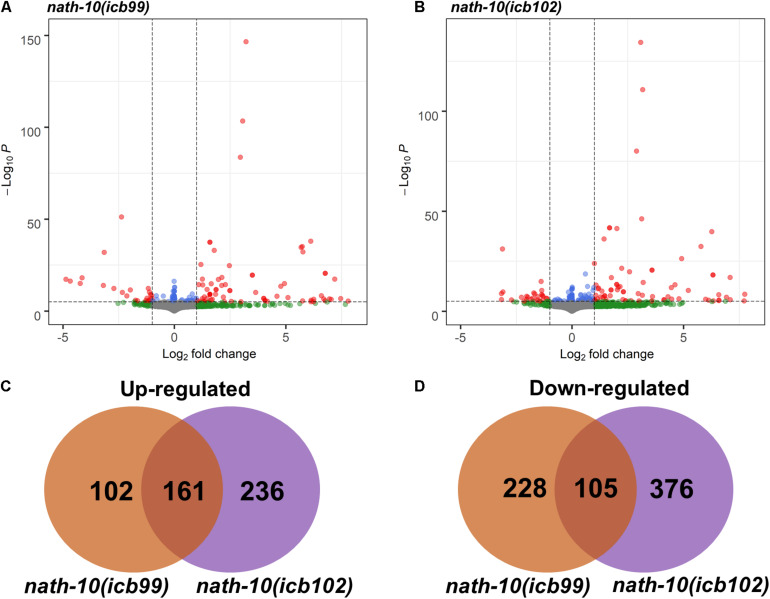
RNA-seq analysis of *nath-10* mutants reveals differentially expressed genes. **(A,B)** Volcano plots displaying summary of *nath-10*(*icb99)* and *nath-10(icb102)* changes in comparison to N2 animals. Green dots indicate > 1.2-fold change, blue dots < 0.01 adjusted *p*-value and red dots > 1.2-fold change and < 0.01 adjusted *p*-value. **(C)** Overlap of significantly differentially upregulated genes in *nath-10*(*icb99)* and *nath-10(icb102)* alleles. **(D)** Overlap of significantly differentially downregulated genes in *nath-10*(*icb99)* and *nath-10(icb102)* alleles.

The human homolog of *nath-10*, NAT10, has recently been shown to catalyze mRNA acetylation of the N4-acetlycytidine, and loss of this acetylation was shown to decrease mRNA stability and translation efficiency (Arango et al., 2018). To study the molecular basis of seam cell number variability, we carried out RNA sequencing on *nath-10* mutants in comparison to wild-type animals. We synchronized animals and harvested them for RNA extraction after 27 h (L2 stage) with three biological replicates for each strain. RNA transcripts were then mapped to the *C. elegans* mRNA transcriptome using Kallisto (Bray et al., 2016) and analyzed using DESeq2 (Anders and Huber, 2010). While both mutants contain an identical *nath-10* mutation, we reasoned that they may still exhibit differences due to the independent origin of the two alleles (one from EMS mutagenesis and the other from CRISPR-mediated genome editing), so we decided to treat them separately for subsequent analysis. Both strains containing *nath-10* alleles displayed differentially regulated genes with significant overlap and could be separated from wild-type animals using principal component analysis (Figures 3A–D and Supplementary Figure 4A). We found that phenotype enrichment analysis of the overlapping genes showed enrichment for epidermal development terms such as “epithelial development variant” and “blistered,” or other terms related to previously known developmental and cellular functions of *nath-10* in fertility and nuclear morphology (Shen et al., 2009; Supplementary Figures 4B,C). To identify specific candidates that may influence the seam cell phenotype, we compared these overlapping transcripts to seam cell specific transcripts identified in a recent single cell RNA-seq analysis of *C. elegans* at L2 (Cao et al., 2017). We found that 67 out of 161 genes in the overlap appeared to be expressed in seam cells and these did not show significant enrichment for any GO terms. Furthermore, we prioritized candidates from the differentially expressed genes based on their seam cell expression and putative connection to other core seam cell genes to test their interaction with *nath-10* using an RNAi screen. However, we found no enhancement or suppression of the seam cell number variance phenotype in these treatments (Supplementary Figure 5).

**FIGURE 4:**
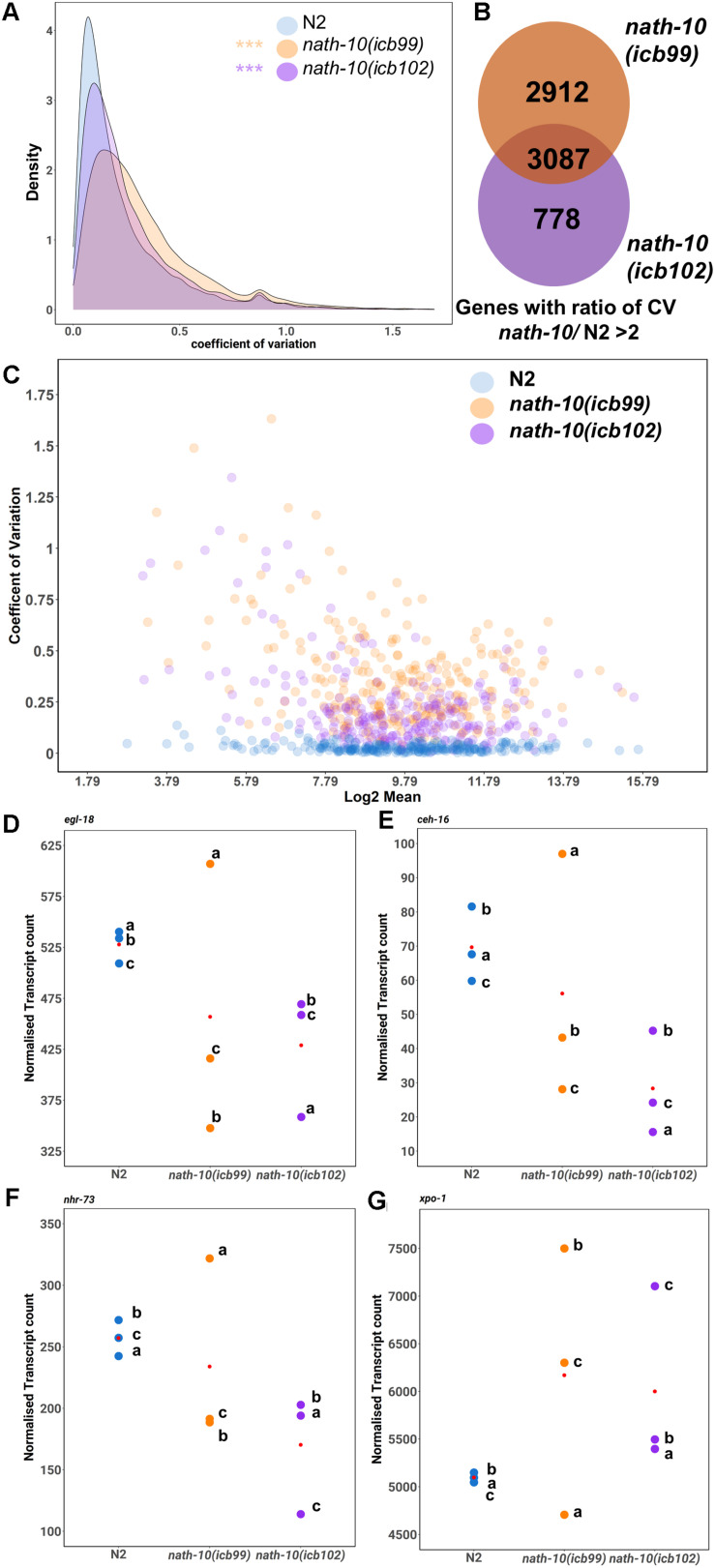
RNA-seq analysis reveals changes in transcript variance at the population level. **(A)** Density plots of coefficient of variation (CV) in *nath-10* mutants and N2. Both *nath-10* alleles show significant change in the distribution of the coefficient of variation compared to N2, with a trend toward an increase in transcript variability (Kolmogorov-Smirnov test, ***p < 0.001). **(B**) Venn diagram displaying the overlap of genes that display a > 2 ratio of CV when compared to N2 for *nath-10*(*icb99)* and *nath-10(icb102)* mutants. Both alleles show a 50% overlap of the most variable genes and this overlap includes core seam cell transcription factors *ceh-16*, *egl-18*, and *nhr-73.* **(C)** Coefficient of variation plotted against the log2 normalized transcript counts from RNA-seq analysis for seam cell-enriched genes in *nath-10* alleles and wild type. **(D–G)** Normalized transcript counts plotted per replicate (a, b, c) for N2, *nath-10(icb99)* and *nath-10(icb102)* showing *egl-18*, *ceh-16*, *nhr-73* and *xpo-1* expression. Note that both *nath-10* alleles display a great spread of normalized transcript count compared to the N2 replicates and the variance is not driven consistently by the same replicate for each gene.

We then decided to explore the possibility that a change in mRNA transcript variance may associate with the variable seam cell number phenotype in the *nath-10* mutant population. Single animal RNA-seq or cell sorting in the epidermis remain experimentally challenging, so we used our RNA-seq data to assess whether a change in transcript variance could be revealed at the population level among replicates. To this end, we calculated the coefficient of variation (CV) for all mapped transcripts that had greater than 5 read counts in two or more of all replicates. While this method would not be able to uncover animal-to-animal differences in variability, we reasoned that it may be possible to uncover population-to-population differences in transcript variance that are beyond what is seen in wild-type populations. Interestingly, we found that both *nath-10* alleles had a significant increase in the average CV compared to N2 (Figure 4A), suggesting that transcript variance is increased.

To identify gene candidates that may relate to the variable seam cell number phenotype, we found common genes between the two *nath-10* alleles that showed higher than twofold increase in transcript variance compared to N2. The overlap contained more than 50% of variable transcripts for each strain (Figure 4B). We compared transcripts that had the highest shift in variance level in our analysis to a seam cell-enriched transcript set (Cao et al., 2017). Interestingly, more than 40% (1,257 transcripts out of 3,087) of the variable genes in the overlap were found to be enriched in the seam cells. Furthermore, this overlap included some transcription factors of the core seam cell gene network, such as *egl-18*, *ceh-16*, and *nhr-73*. For example, *egl-18* was in the top 250 of most variable genes together with other genes including *xpo-1*, for which the human homolog (XPO1/CRM1) has been shown to be linked to NAT10 levels in human cells (Zhang et al., 2014; Figures 4C–E). Plotting of the mean transcript number for each replicate indicates that the increase in variance in *nath-10* mutants was not driven by the same underlying pattern for each selected gene, such as a specific replicate being consistently the outlier (Figures 4D–G).

To further investigate whether the transcript variance shown by *nath-10* loss-of function was due to animals being sensitized to environmental conditions, we isolated functional subsets of genes that display changes during aging, temperature shifts or exposure to stress and compared the CV pattern between N2 and *nath-10* mutants. Interestingly, we found that the *nath-10* mutants did not appear different in transcript variance compared to N2 animals for these subsets of genes (Supplementary Figures 6A–C). Furthermore, analysis of highly variable genes found in N2 did not display increased levels of variance beyond their wild-type level of variation in *nath-10* alleles (Supplementary Figure 6D), indicating that there is no evidence to suggest that *nath-10* alleles were more sensitive to environmental variation. Taken together, these results suggest that *nath-10* loss-of-function increases transcript variability across populations for a number of genes expressed in seam cells.

### Wnt Pathway Activation Is Disrupted When *nath-10* Function Is Impaired

We then sought to uncover a potential seam cell target that may underlie some of the seam cell defects observed in *nath-10* mutants. We focused on the GATA transcription factor *egl-18* (Koh and Rothman, 2001; Gorrepati et al., 2013) identified in the RNA-seq analysis above, which has been implicated in seam cell development as a direct target of Wnt signaling (Gorrepati et al., 2013).

**FIGURE 5:**
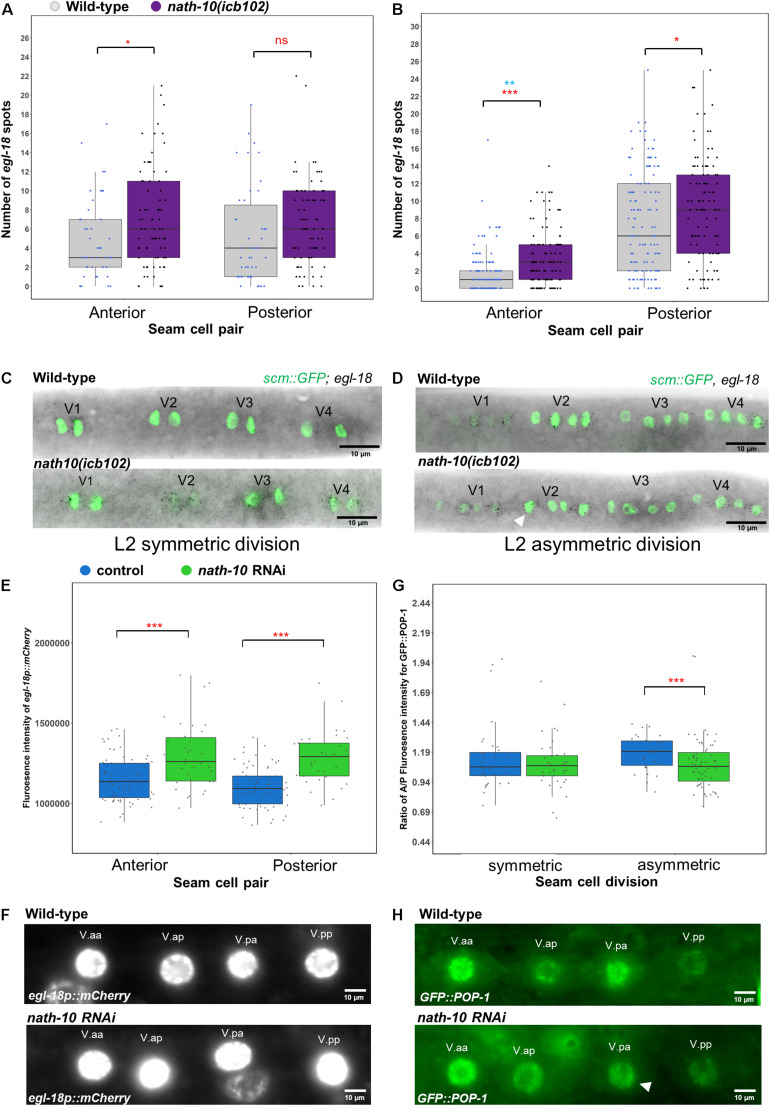
*nath-10* loss-of-function leads to changes in Wnt signaling and activation. **(A,B)** smFISH counts of *egl-18* in L2 symmetric **(A)** and asymmetric **(B)** cell division of V1-V4 lineage cells in wild type (gray) and *nath-10(icb102)* (purple). Note that *nath-10(icb102)* animals show a significant increase in mRNA transcript counts in anterior V lineage daughters [*t*-test, *p < 0.05, wild type *n* = 46, *nath-10(icb102) n* = 78 cells]. **(B)** smFISH counts of *egl-18* in L2 asymmetric V cell lineage division. Anterior and posterior V lineage cells in *nath-10(icb102)* show a significant increase in average mRNA count and variability [*t*-test, ***p < 0.001 in red, wild type *n* = 129, *nath-10(icb102) n* = 106 cells and Levene’s test **p < 0.01, blue]. **(C,D)** Representative images of *egl-18* smFISH in wild type and *nath-10(icb102)* animals at L2 symmetric and asymmetric division stage. **(E)** Quantification of fluorescence intensity from the *egl-18::mCherry* reporter in the nuclei of V lineage seam cell pairs (V.aa/V.ap and V.pa/V.pp) at the asymmetric L2 division. *egl-18::mCherry* is significantly increased in the anterior and posterior V lineage seam cell daughters in animals exposed to *nath-10* RNAi compared to empty vector control (***p < 0.001, *t*-test, wild type *n* = 69, *nath-10 RNAi n* = 34). **(F)** Representative images for *egl-18p::mCherry* in seam cell nuclei pairs (V.aa/V.ap and V.pa/V.pp) at L2 asymmetric division stages for *nath-10* RNAi exposed animals and empty vector control. **(G)** Quantification of anterior/posterior cell ratio of GFP::POP-1 at the symmetric and asymmetric L2 division in V lineage seam cell pairs (V.aa/V.ap and V.pa/V.pp). A significant decrease in this ratio was found when animals were exposed to *nath-10* RNAi (***p < 0.001, *t*-test). **(H)** Representative GFP::POP-1 images in control and *nath-10* RNAi animals, arrowhead indicates representative anterior V lineage cell with lower expression of GFP::POP-1 (wild type *n* = 31, *nath-10 RNAi n* = 65).

To test whether *egl-18* expression is altered in the *nath-10* mutant background, we carried out comparative *egl-18* smFISH at the L2 symmetric and asymmetric cell divisions, focusing on V seam cell lineages where most of the developmental errors occur. We found that *egl-18* levels were mildly increased in V lineage seam cells during the symmetric and asymmetric cell division (Figures 5A–D). We also found that *egl-18* mRNA levels were significantly increased and more variable in anterior seam cell daughters, which normally differentiate in wild type following the asymmetric L2 division (Figures 5B,D). To further confirm these changes in *egl-18* expression, we analyzed animals carrying an *egl-18* transcriptional reporter, *egl-18p::mCherry*. In concurrence with the mRNA changes observed by smFISH, we found an increase in the *egl-18* reporter expression and variability at the asymmetric division during the L2 stage when animals are exposed to *nath-10* RNAi compared to empty vector control (Figures 5E,F). Since *egl-18* is known to play a role in seam cell fate acquisition (Gorrepati and Eisenmann, 2015), the increase in *egl-18* expression in *nath-10* mutants may be indicative of ectopic Wnt target activation and adoption of seam cell fate. This effect is likely to explain seam cell gains, which are somewhat more frequent than seam cell losses in *nath-10(icb102)* mutant populations (91 errors in 335 animals out of which 67 are seam cell gains and 24 are seam cell losses).

As *egl-18* expression is a consequence of Wnt pathway activation, we sought to understand if the distribution of its upstream activator POP-1 is also changed upon perturbing *nath-10* levels. Following wild-type asymmetric seam cell division, POP-1 levels are high in the anterior daughter nucleus and low in the posterior daughter nucleus. In the anterior daughter, high levels of POP-1 are thought to lead to a suppression of Wnt targets, while low levels of POP-1 in the posterior daughter result in Wnt target activation (Lin et al., 1998; Rocheleau et al., 1999; Phillips et al., 2007; Gleason and Eisenmann, 2010). We measured GFP::POP-1 fluorescent intensity in the nucleus of anterior and posterior V lineage cells following the L2 symmetric and asymmetric seam cell divisions in *nath-10* RNAi exposed animals and control treatments. We found a lower ratio of anterior to posterior nuclear POP-1 levels in *nath-10* RNAi animals compared to control treated animals at the asymmetric division stage, and no significant difference at the symmetric division stage (Figures 5G,H). This finding indicates that a number of V cell lineage daughters in *nath-10* RNAi treated animals appear to have more equal POP-1 levels in the nucleus. These results are in line with the increase in *egl-18* expression seen in anterior V lineage daughters and suggest that *nath-10* loss-of-function may interfere with the pattern of Wnt pathway activation through changes in the cellular distribution of POP-1.

## Discussion

Through an unbiased genetic screen followed up by a multifaceted validation approach, we identify here a new role for *nath-10* in modulating epidermal stem cell number in *C. elegans*. The N-acetyltransferase *nath-10* is the homolog of the human NAT10, which is known to localize in the nucleolus and is shown to be involved in the regulation of telomerase activity, tRNA transcription and cytokinesis via acetylation of microtubules (Chi et al., 2007; Shen et al., 2009; Cai et al., 2017; Sleiman and Dragon, 2019). In *C. elegans*, the molecular function of *nath-10* has not been previously studied. However, it is known that cryptic genetic variation in *nath-10* has been selected in the reference strain N2 for optimal growth under laboratory conditions (Duveau and Félix, 2012). It is conceivable that *nath-10*, like its human homolog, may have multiple interaction partners and be involved in controlling a number of cellular processes. Therefore, impairment of *nath-10* function may have broad phenotypic consequences at the individual and population level.

We report that a partial loss-of-function of *nath-10* increases transcript variance across populations. While it is difficult to completely rule out technical variability as a factor contributing to the higher variance observed in *nath-10* replicates, we think that this is unlikely to be the case for multiple reasons. For example, we did not observe variable growth in the *nath-10* mutant background and we did not find a specific biological replicate being consistently an outlier in the gene expression analysis possibly reflecting variability or error while conducting these experiments. Evidence in yeast suggests that regulators of robustness to stochastic variation can also influence the robustness to environmental variation (Lehner, 2010). It is therefore possible that *nath-10* mutations may also sensitize the animals to environmental variation. However, we found that increase in transcript variance does not apply to several temperature or stress-related genes. Furthermore, noisy genes in wild type, which are predicted to be more responsive to environmental variation (Lehner and Kaneko, 2011), do not appear to be hypervariable in *nath-10* mutants. One way to explain mRNA transcript variability is through transcript stability, which can readily explain changes in gene expression (Ross, 1995). A recent study showed that the human NAT10 may be responsible for mRNA stability and translation efficiency through N4-actelylation of cytidine present on mRNA transcripts (Ito et al., 2014; Arango et al., 2018). Therefore, it is possible that a change in the ability of NATH-10 to carry out this acetylation may change the stability of mRNA transcripts. Interestingly, the mutation recovered from our screen falls outside the known domains of the protein and therefore may cause a mild structural change, which could compromise the ability of NATH-10 to perform its function. Proline residue mutations have been shown to affect protein structure and subsequent binding efficiency (Davis et al., 2018) so this amino acid change or a decrease in NATH-10 levels may lower acetylation and therefore destabilize mRNA transcripts. It remains unclear at this point what is the biological significance of transcript variance across populations for the variable seam cell number phenotype, but a key prediction from this work is that molecular variation may also occur within single animals in the *nath-10* mutant population and this may contribute to the destabilization of seam cell development.

NAT10 has also been implicated in the rapid cellular aging disease Hutchinson-Gilford progeria (HGP). A recent study showed that cells affected by HGP, chemical inhibition of NAT10 through application of a small molecule caused the rapid aging of cells to be reversed (Larrieu et al., 2018). It was further shown that an interaction with the microtubule network and the disruption of a nuclear import pathway and nuclear pore complexes was responsible for this phenotype. During seam cell division, the import and export of factors in and out of the nucleus is crucial for correct cell fate patterning. This is exemplified by the nuclear accumulation of the TCF/LEF factor POP-1, which changes in Wnt responding cells and was found to be perturbed upon *nath-10* knockdown. It is possible that impairment of *nath-10* may interfere with nuclear import and export pathways, as seen for example in the variability of expression of the exportin 1 homolog *xpo-1*, which may further disrupt protein localization during stem cell patterning.

Seam cell division patterns and polarity are predominantly under the control of Wnt signaling (Takeshita and Sawa, 2005; Wildwater et al., 2011; Yamamoto et al., 2011; Gorrepati et al., 2013; van der Horst et al., 2019). We describe a new link between NATH-10 and Wnt signaling, based on the impaired distribution of a Wnt effector and subsequent activation of a Wnt pathway target in *nath-10* RNAi treated animals. Strikingly, anterior V cell daughters following the second L2 division displayed an increase in average number and variance of *egl-18* mRNA transcripts. These observations were reproduced with a transcriptional *egl-18::mCherry* marker, suggesting that changes in the *nath-10* background reflect transcriptional activity of *egl-18*, likely through changes in upstream regulators. This is consistent with the changes found in POP-1 distribution in *nath-10* RNAi treated animals. POP-1 is the TCF/LEF factor that is responsible for activating Wnt target genes including *egl-18*, and seam cell fate through asymmetric nuclear accumulation (Lin et al., 1995; Rocheleau et al., 1999; Mizumoto and Sawa, 2007; Phillips et al., 2007; Gorrepati et al., 2013). It is of note that POP-1 requires acetylation at three specific sites to increase its nuclear export efficiency (Gay et al., 2003). NAT10 has been shown to have a role in regulating nuclear export machinery and is able to acetylate multiple substrates, so it is therefore possible that *nath-10* may play a direct role in the control of POP-1 or other core Wnt components (Larrieu et al., 2018). Future work is required to be able to understand how NATH-10 mechanistically interacts with the Wnt signaling pathway to facilitate robust seam cell fate patterning.

## Data Availability Statement

The datasets presented in this study can be found in online repositories. The names of the repository/repositories and accession number(s) can be found in the article/Supplementary Material.

## Author Contributions

MH carried out the majority of experiments and data analysis. DK developed the hairpin construction platform and contributed to mutation mapping. VS advised on gene expression variability analysis. MB supervised the work. MH, DK and MB wrote the manuscript. All authors contributed to the article and approved the submitted version.

## Conflict of Interest

The authors declare that the research was conducted in the absence of any commercial or financial relationships that could be construed as a potential conflict of interest.

## Supplementary Material

Supplementary Figure 1 Design for hairpin construction and validation of the system in the epidermis. **(A)** A schematic of the hairpin RNAi construct. The final construct carries restriction sites flanking the promoter site, to allow for insertion of any promoter of interest, such as a seam cell and hypodermis-specific promoter. It also carries two entry sites (forward and reverse) for the insertion of the inverted fragments, flanking intron 5 of *srf-3* isoform a, followed by a 3′ UTR. The insertion sites contain inverted repeats of recognition sites for the Type IIS restriction enzymes *Bpi*I in the forward entry site and *Esp*3I in the reverse. These enzymes cut asymmetrically 2–6 bp away from the recognition sequence creating 5′ 4 bp long overhangs. The sequences cleaved for both enzymes create incompatible non-palindromic overhangs, so that a fragment with compatible overhangs can be inserted in the two sites in opposite orientations. Transgenic animals in all panels carry *myo-2p:dsRED* marker. **(B)** Seam cell specific knockdown of targets was validated by creating a hairpin against GFP and expressing this under the *srf-3* promoter. Note that *scm:GFP* was strongly reduced in seam cells in transgenic animals (left panel). A hypodermal specific GFP hairpin showed strong knockdown of *dpy-7p:GFP* in hypodermal cells (middle panel) but did not affect *scm:GFP* expression in seam cells.

Supplementary Figure 2 Increase of seam cell number variance in *nath-10(icb102)* mutants is reproducible. Seam cell counts for wild type and *nath-10(icb102)* animals carried out on separate days show similar average seam cell counts and reproducibly significant increase in variability (Levene’s test *^∗∗∗^p* < 0.001).

Supplementary Figure 3 Characterization of *nath-10* mutant phenotypes and expression outside the seam cells. **(A)** Brood size assay for N2, *nath-10(icb102)* and *nath-10(icb99)*. There was a significant decrease in the number of progeny for both strains carrying *nath-10* alleles (*t*-test, ^∗∗∗^*p* < 0.001, red), but there was no significant difference in the variability of progeny number (*P* > 0.05, Levene’s test, blue). **(B)** Proportion of L4 animals in N2 and *nath-10* alleles at defined time intervals. No significant delay in developmental timing was observed for the *nath-10* alleles compared to wild-type animals. **(C)** Wild-type animals show *nath-10* expression by smFISH within the gonad, outlined in yellow, at the late L4 stage.

Supplementary Figure 4 PCA of all replicates used in the RNA-seq analysis and GO term analysis of differentially expressed genes. **(A)** PCA analysis of samples used for RNA-seq analysis indicates *nath-10* and wild-type replicates can be separated on the PC1 axis. **(B,C)** GO analysis of upregulated genes **(A)** or downregulated genes **(B)**. Significance was calculated using a hypergeometric probability test and corrected by the Benjamini-Hochberg step-up algorithm with a *q-value* threshold of 0.1.

Supplementary Figure 5 RNAi screen on candidate genes to test interaction with the *nath-10* seam cell phenotype. **(A–E)** Seam cell counts for selected candidates showed no significant interaction with the *nath-10(icb102)* seam cell number variability. Candidate genes for the RNAi screen were selected as top on the list of differentially expressed genes using the following filtering criteria: fold change, seam cell expression, seam cell phenotype and predicted interaction with either core seam cell network genes (*ceh-16*, *egl-18* and *elt-1*) or *nath-10*.

Supplementary Figure 6 Analysis of variance within functional subsets of genes for N2 and *nath-10* alleles. **(A–C)** Plots of the coefficient of variation against the log2 average normalized transcript count for N2 and *nath-10* alleles. **(D)** Genes with the highest coefficient of variation in N2 were extracted from the RNA-seq data. Note that *nath-10* alleles do not show variation beyond what is found in N2 in all of these cases. Functional subsets were generated from the following gene expression clusters: WBPaper00038118:stress_upregulated_N2 **(A)**, WBPaper0032062:age_regulated_genes **(B)**, and WBPaper00039792:TempShift **(C)**.

Supplementary Table 1 List of strains used in this study.

Supplementary Table 2 List of plasmids, oligos and smFISH probes used in the study.

Supplementary Table 3 RNA-seq analysis and gene set comparisons.
