# Supplementary figures and images for: Phenotypic Robustness of Epidermal Stem Cell Number in C. elegans Is Modulated by the Activity of the Conserved N-acetyltransferase nath-10/NAT10
Source: Front Cell Dev Biol. 2021 May 18;9:640856. doi: 10.3389/fcell.2021.640856 (PMC8168469; doi:10.3389/fcell.2021.640856)

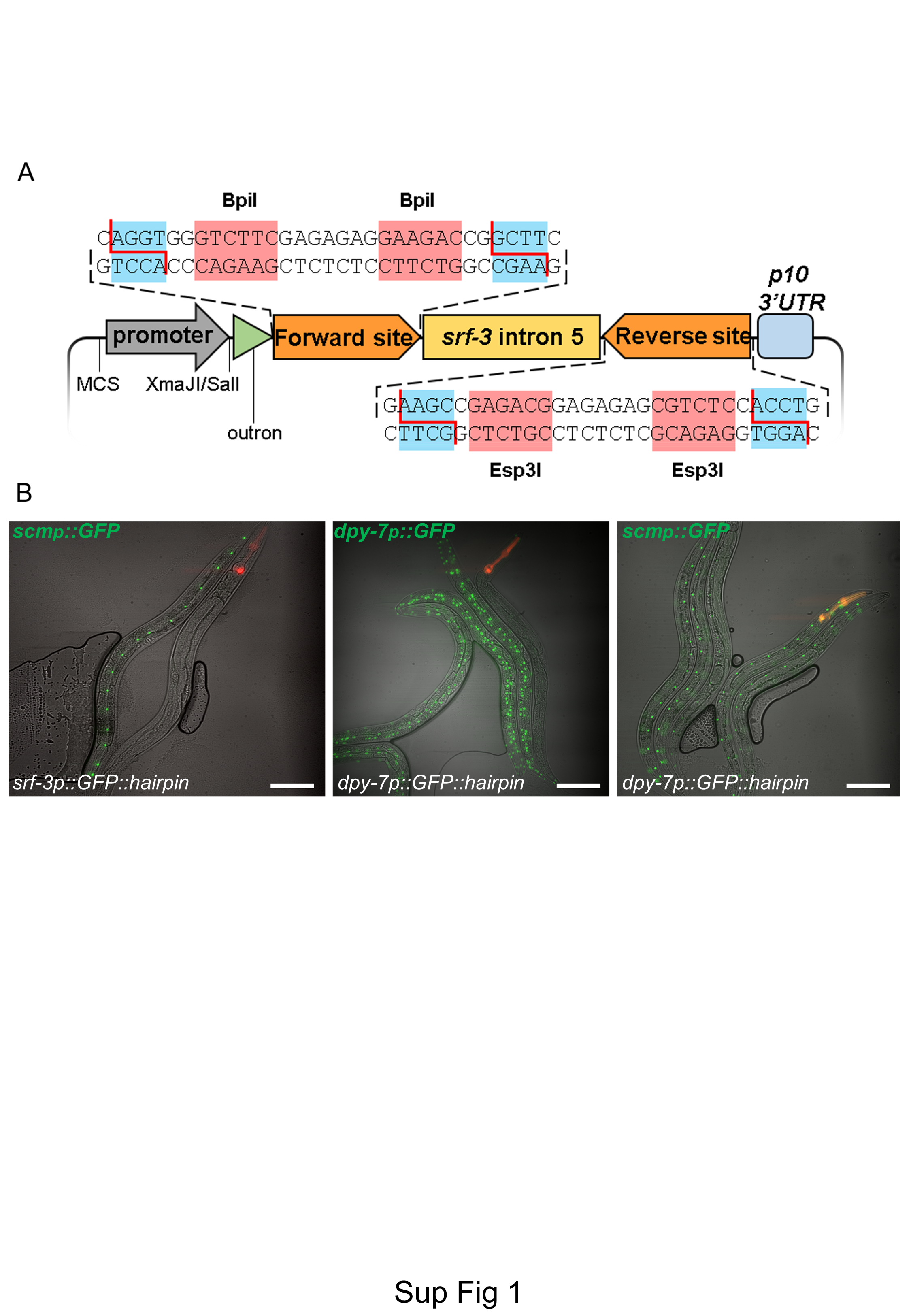

Supplement: Supplementary Figure 1 — Design for hairpin construction and validation of the system in the epidermis. (A) A schematic of the hairpin RNAi construct. The final construct carries restriction sites flanking the promoter site, to allow for insertion of any promoter of interest, such as a seam cell and hypodermis-specific promoter. It also carries two entry sites (forward and reverse) for the insertion of the inverted fragments, flanking intron 5 of srf-3 isoform a, followed by a 3′ UTR. The insertion sites contain inverted repeats of recognition sites for the Type IIS restriction enzymes BpiI in the forward entry site and Esp3I in the reverse. These enzymes cut asymmetrically 2–6 bp away from the recognition sequence creating 5′ 4 bp long overhangs. The sequences cleaved for both enzymes create incompatible non-palindromic overhangs, so that a fragment with compatible overhangs can be inserted in the two sites in opposite orientations. Transgenic animals in all panels carry myo-2p:dsRED marker. (B) Seam cell specific knockdown of targets was validated by creating a hairpin against GFP and expressing this under the srf-3 promoter. Note that scm:GFP was strongly reduced in seam cells in transgenic animals (left panel). A hypodermal specific GFP hairpin showed strong knockdown of dpy-7p:GFP in hypodermal cells (middle panel) but did not affect scm:GFP expression in seam cells. [file Image_1.TIF]

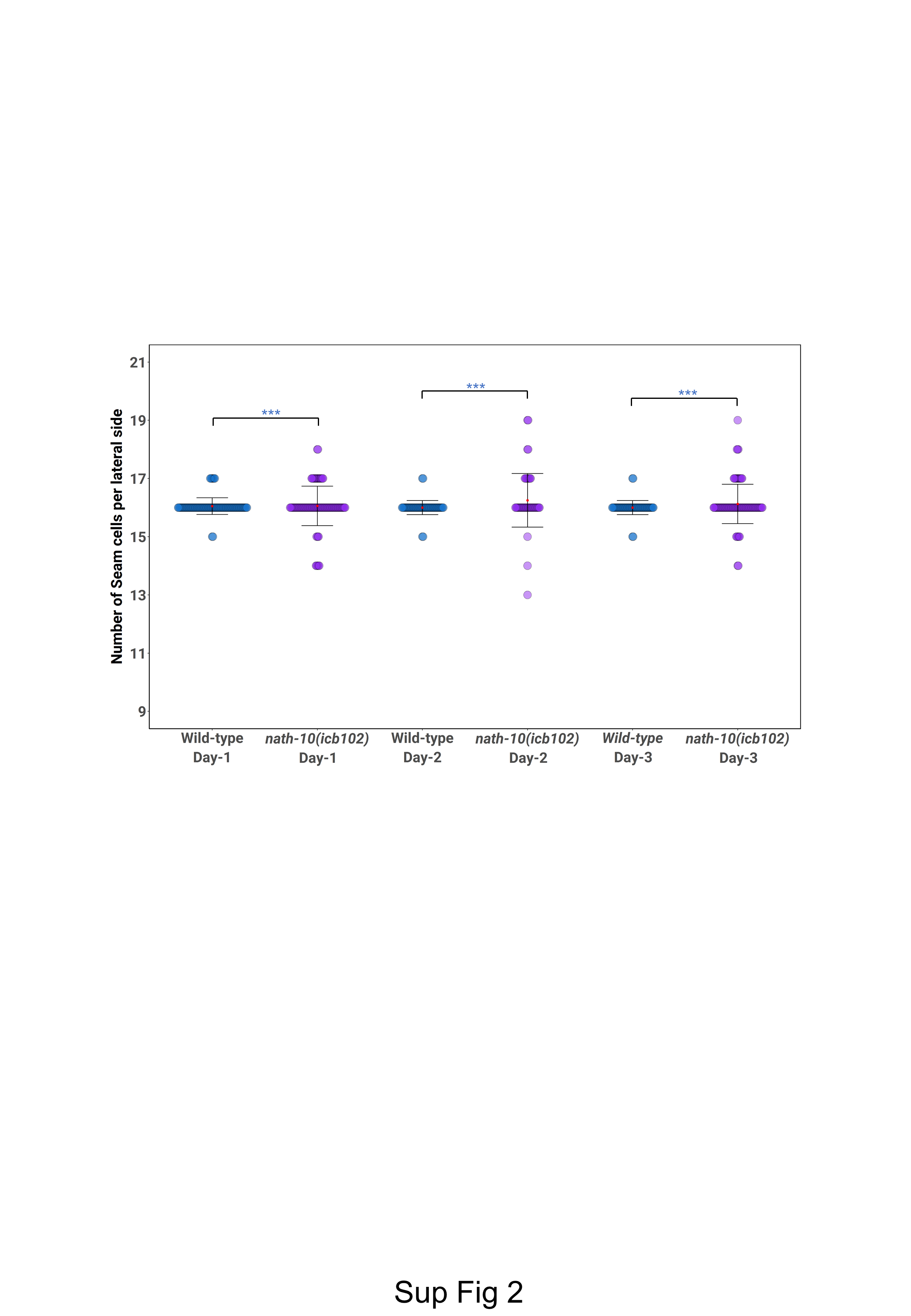

Supplement: Supplementary Figure 2 — Increase of seam cell number variance in nath-10(icb102) mutants is reproducible. Seam cell counts for wild type and nath-10(icb102) animals carried out on separate days show similar average seam cell counts and reproducibly significant increase in variability (Levene’s test ∗∗∗p < 0.001). [file Image_2.TIF]

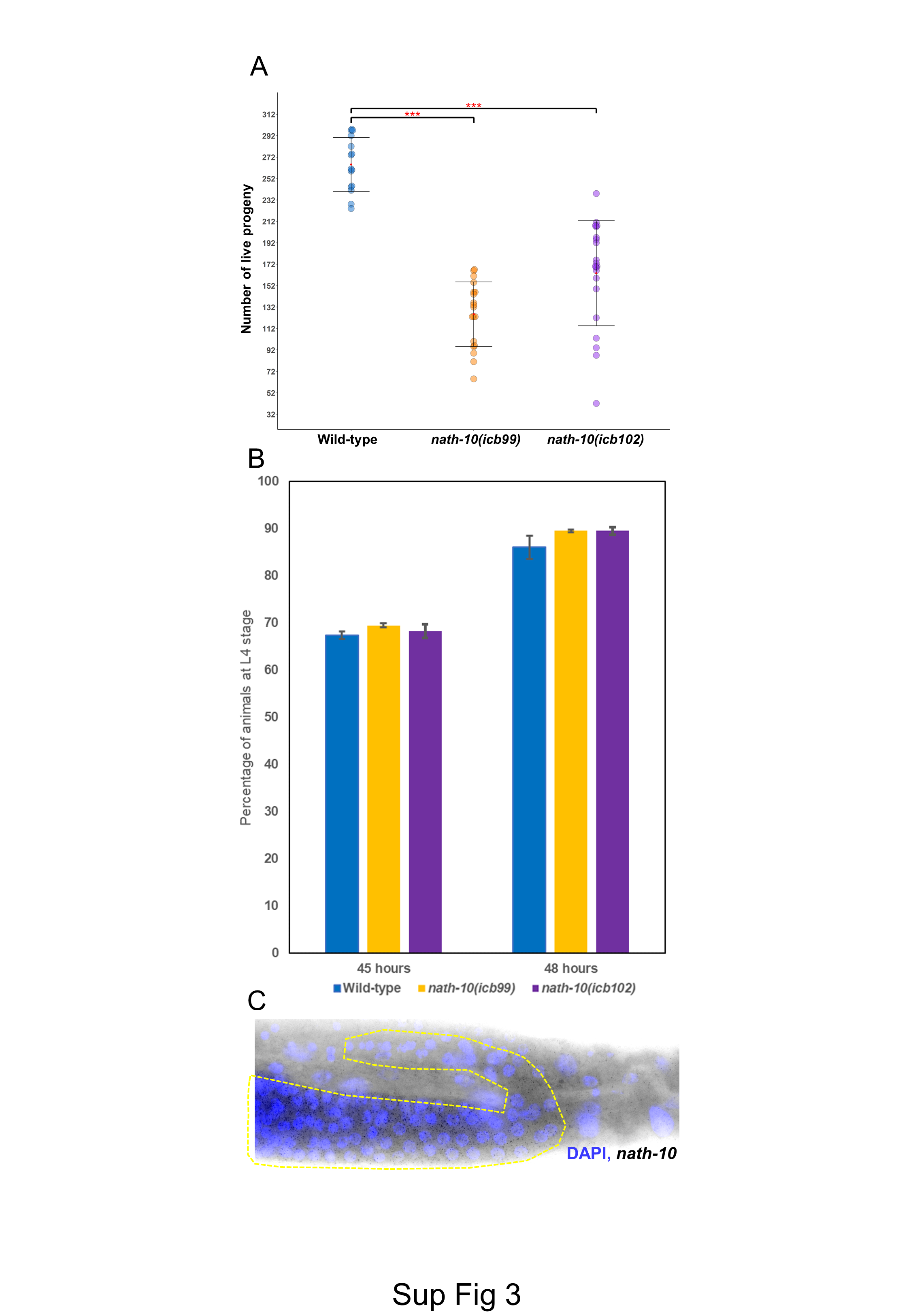

Supplement: Supplementary Figure 3 — Characterization of nath-10 mutant phenotypes and expression outside the seam cells. (A) Brood size assay for N2, nath-10(icb102) and nath-10(icb99). There was a significant decrease in the number of progeny for both strains carrying nath-10 alleles (t-test, ∗∗∗p < 0.001, red), but there was no significant difference in the variability of progeny number (P > 0.05, Levene’s test, blue). (B) Proportion of L4 animals in N2 and nath-10 alleles at defined time intervals. No significant delay in developmental timing was observed for the nath-10 alleles compared to wild-type animals. (C) Wild-type animals show nath-10 expression by smFISH within the gonad, outlined in yellow, at the late L4 stage. [file Image_3.TIF]

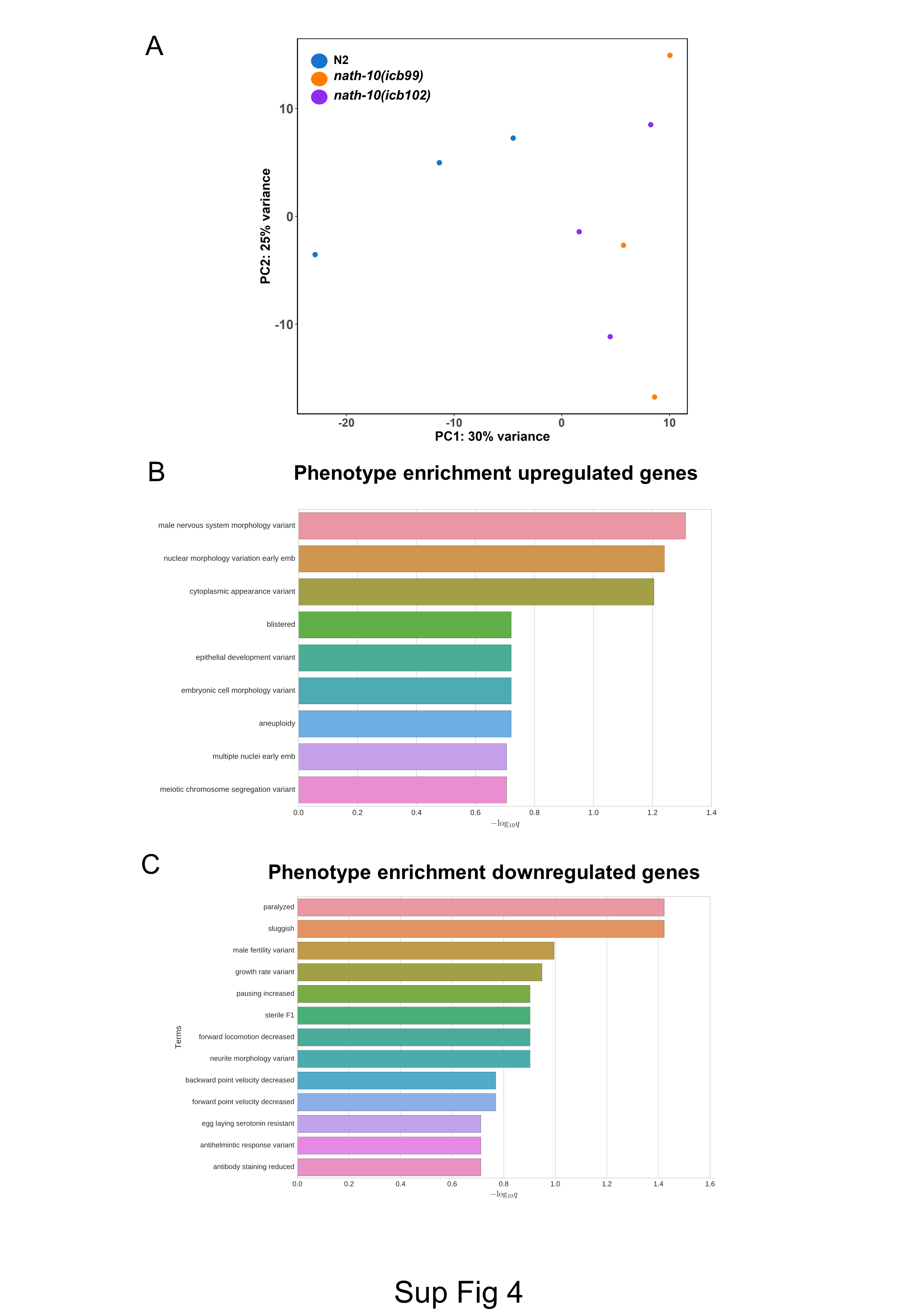

Supplement: Supplementary Figure 4 — PCA of all replicates used in the RNA-seq analysis and GO term analysis of differentially expressed genes. (A) PCA analysis of samples used for RNA-seq analysis indicates nath-10 and wild-type replicates can be separated on the PC1 axis. (B,C) GO analysis of upregulated genes (A) or downregulated genes (B). Significance was calculated using a hypergeometric probability test and corrected by the Benjamini-Hochberg step-up algorithm with a q-value threshold of 0.1. [file Image_4.TIF]

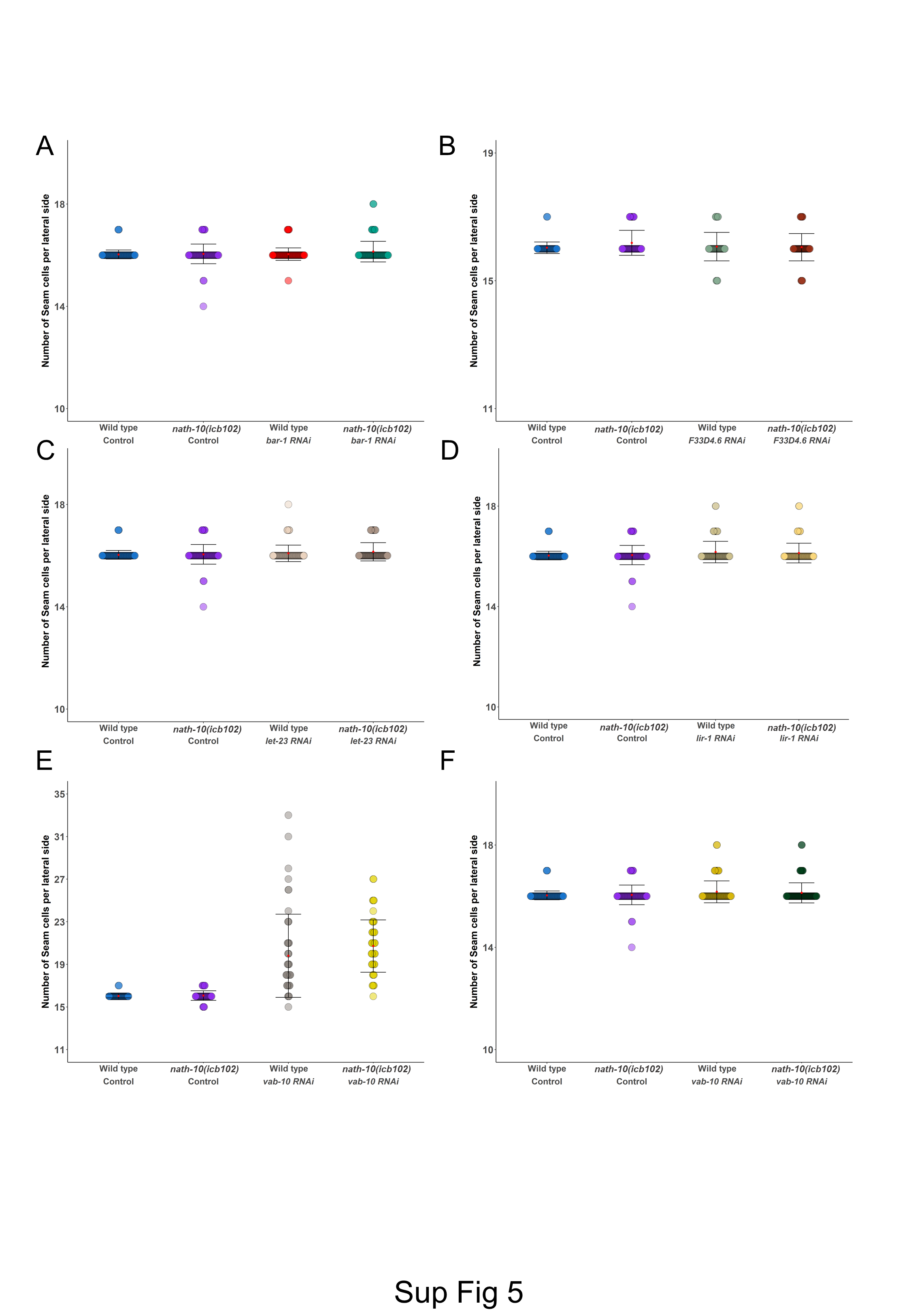

Supplement: Supplementary Figure 5 — RNAi screen on candidate genes to test interaction with the nath-10 seam cell phenotype. (A–E) Seam cell counts for selected candidates showed no significant interaction with the nath-10(icb102) seam cell number variability. Candidate genes for the RNAi screen were selected as top on the list of differentially expressed genes using the following filtering criteria: fold change, seam cell expression, seam cell phenotype and predicted interaction with either core seam cell network genes (ceh-16, egl-18 and elt-1) or nath-10. [file Image_5.TIF]

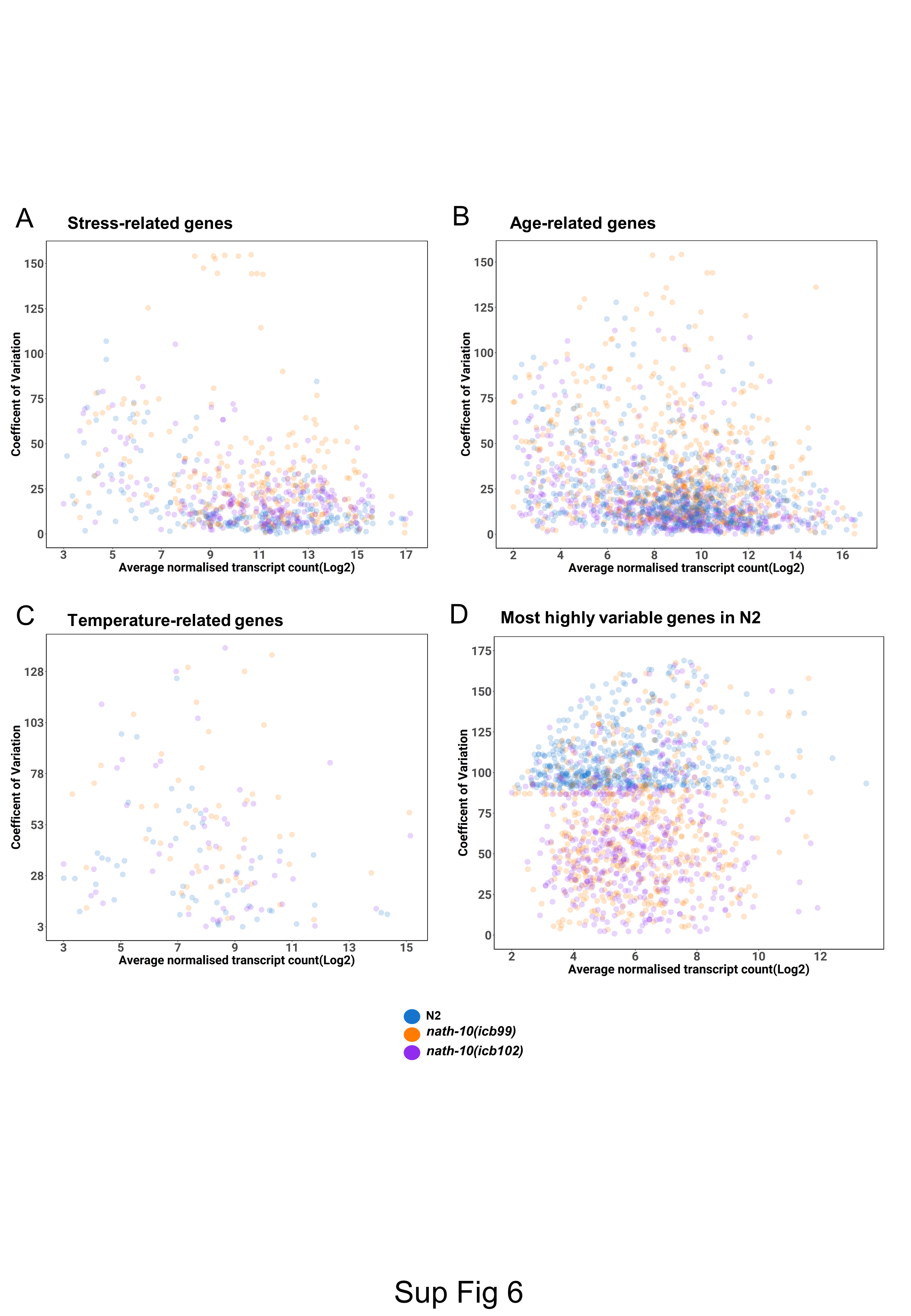

Supplement: Supplementary Figure 6 — Analysis of variance within functional subsets of genes for N2 and nath-10 alleles. (A–C) Plots of the coefficient of variation against the log2 average normalized transcript count for N2 and nath-10 alleles. (D) Genes with the highest coefficient of variation in N2 were extracted from the RNA-seq data. Note that nath-10 alleles do not show variation beyond what is found in N2 in all of these cases. Functional subsets were generated from the following gene expression clusters: WBPaper00038118:stress_upregulated_N2 (A), WBPaper0032062:age_regulated_genes (B), and WBPaper00039792:TempShift (C). [file Image_6.TIF]
